# Microstructured thermo-responsive double network granular hydrogels†

**DOI:** 10.1039/d5ma00511f

**Published:** 2025-06-16

**Authors:** Alexandra Thoma, Reece Whatmore, Esther Amstad

**Affiliations:** a Soft Materials Laboratory, Institute of Materials École Polytechnique Fédérale de Lausanne 1015 Lausanne Switzerland esther.amstad@epfl.ch

## Abstract

Many hydrogels respond to external stimuli such as changes in temperature, pH, or salt concentrations by changing their degree of swelling, and hence mechanical properties, rendering them attractive actuators. Unfortunately, response rates of many of these hydrogels are limited because they rely on the diffusion of water, which is relatively slow within the gel. Here, we introduce thermo-responsive granular hydrogels which combine accelerated response rates with load-bearing properties. To accelerate the response to temperature changes, we formulate poly(*N*-isopropylacrylamide) (PNIPAM) microgels with connected pores by leveraging phase separations. To impart the porous hydrogel load-bearing properties, we formulate them as thermo-responsive double network granular hydrogels (TDNGHs). We demonstrate that the granular structure combined with the open micropores located within the microfragments increase the response-rate of these gels 3-fold compared to that of bulk counterparts. Moreover, the granular material exhibits 18-fold enhanced work of fracture compared to the bulk. The granular structure adds an additional benefit: it renders them 3D printable. We co-process thermo-responsive hydrogels with a non-responsive counterpart to fabricate a bilayer, which lifts up to 85% of its weight if heated and 3D print a butterfly as a bilayer structure that bends its wings when exposed to elevated temperatures.

## Abstract 2

Thermo-responsive double network granular hydrogels made of microgels with open micrometer-sized pores can lift up to 85% of their own weight and respond up to 3-fold faster to changes in temperature than bulk counterparts are introduced.

## Graphical abstract

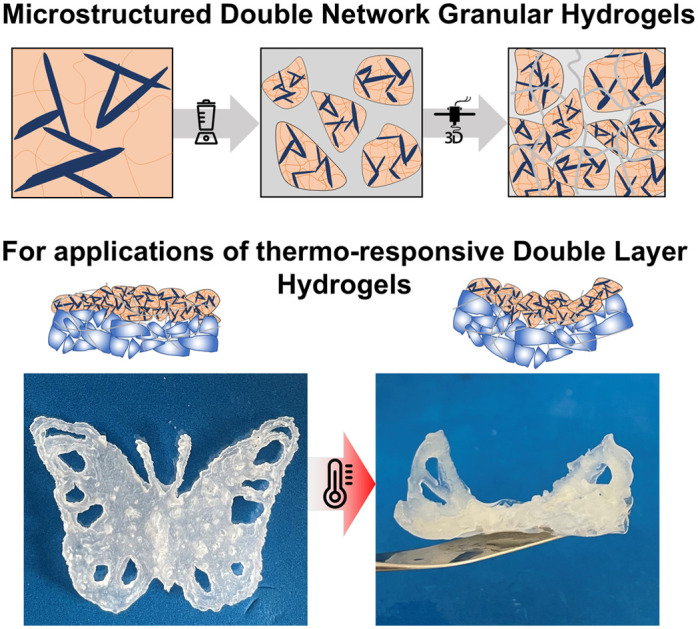

## 1. Introduction

Hydrogels are highly hydrated polymeric networks. Certain hydrogels respond to external stimuli, such as variations in pH, temperature, or light illumination by changing their water content.^1–3^ This feature has been leveraged to activate materials, for example in soft robotics, drug delivery, and sensing.^2,4,5^ A prominent example of a thermo-responsive hydrogel is poly(*N*-isopropylacrylamide) (PNIPAM), which has a lower critical solution temperature (LCST) of around 32 °C.^6^ As a result of its reversible temperature-dependent change in the degree of swelling, PNIPAM has been used to form shape-morphing structures.^3,4,7,8^ To enable controlled morphological changes, such as bending, responsive hydrogels are typically formulated as a bilayer composed of a responsive layer made, for example, of PNIPAM and an inert layer composed of, for example, gelatin,^9^ acrylic acid-acrylamide copolymers^10^ or a metal reinforced alginate.^11^ This strategy has been employed to form a four-arm gripper that closes to capture an object if exposed to elevated temperatures.^12,13^ The temperature-responsiveness of PNIPAM has also been leveraged to trigger the release of drugs,^14,15^ agrochemicals,^16^ and preservatives.^17^ Unfortunately, the response rate of hydrogels to changes in temperature is slow as it relies on water diffusion.^18,19^ It takes typically 10s of seconds to minutes to actuate structures with cross-sections of order mm^2^.^13,20^ The actuation speed of PNIPAM increases if it contains pores and even more, up to 14% s^−1^, if the pores are connected.^21^ Pores with diameters up to 100 μm have been introduced into PNIPAM by freezing to form ice crystals,^22^ incorporating minerals^23^ or emulsion drops^24^ as porogens, exploiting solvent-induced phase separations^21,25–27^ or with microengineering approaches that lead to interconnected microchannels.^28^ Unfortunately, the introduction of pores into PNIPAM hydrogels often reduces their stiffness such that the response rate must be traded off with the mechanical properties.^29^ This trade-off is particularly limiting considering the softness and brittleness of virgin PNIPAM that prevents its use for load-bearing applications already in the virgin, nonporous state.^30^ The mechanical properties of PNIPAM can be improved if formulated as double network hydrogels,^31–33^ co-polymerized with strong polymers,^34^ or if phase separations that lead to polymer-rich domains are leveraged.^35^ For instance, the copolymerization of PNIPAM with acrylic acid increased its toughness because PAA could be reinforced with metal ions.^36^ However, the response rates of these reinforced materials were slow. Moreover, the rheological properties of precursor solutions limit their processing to casting such that only simple, homogeneous structures can be obtained. Formulations that combine fast response rates with load-bearing properties remain to be established.

Here, we introduce 3D printable load-bearing thermo-responsive double network granular hydrogels (TDNGHs), which combine fast response rates with load-bearing properties. TDNGHs are composed of PNIPAM microfragments, which are connected through a 2nd percolating PNIPAM network. To increase the response rates of TDNGHs, we introduce micropores into microfragments by exploring the phase separation between water and dimethylsulfoxide (DMSO).^21,37^ These pores increase the work of fracture of TDNGHs 18-fold without compromising their Young's modulus. In addition, they increase the response rate of TDNGHs three-fold. We leverage the thermo-responsiveness of this material to fabricate a hydrogel bilayer, which deforms if heated such that it can lift 85% of its own weight or push a 3D printed ball with a diameter of 7 mm underwater. We take advantage of the rheological properties of the jammed microfragments to 3D print a butterfly that bends its wings if heated.

## 2. Experimental section/methods

### 2.1. Materials

*N*-Isopropylacrylamide (PNIPAM) (ABCR Chemicals, 218-638-5), acrylamide (AM) (Sigma-Aldrich, A4058), poly(2-acrylamid-2-methyl-1-propanesulfonicacid) (PAMPS) (Sigma-Aldrich, 102131190), *N*,*N*′-methylene bisacrylamide (MBA) (Carl Roth, 7867.1), 2-hydroxy-2-methylpropiophenone used as a photoinitiator (PI) (Sigma-Aldrich, 405655) and dimethylsulfoxide (DMSO) (Carl Poly Roth, 200-664-3) were used as received.

### 2.2. Production of the microfragments

Hydrogels were prepared as bulk hydrogels. An aqueous solution containing 20 wt% NIPAM, 0.5 wt/wt% MBA, and a 1 μL mL^−1^ photoinitiator was mixed and protected from light. For nonporous hydrogels, deionized water was used, for hydrogels possessing small micropores, a mixture of 40 mol% DMSO and 60 mol% water was used and for hydrogels with large pores, a mixture of 60 mol% DMSO and 40 mol% water was used. The aqueous solution was crosslinked with UV light (Camag UV 4 Lamp) for 10 min to trigger the free radical polymerization reaction of the PNIPAM network and for the porous samples the phase separation. The bulk hydrogels were fragmented at room temperature with a blender (Princess Zerkleiner multi) for 5 min to produce microfragments. The microfragments were put into deionized water, sieved with a 500 μm mesh and further filtered with a 100 μm mesh syringe filter to remove large fragments. The microfragments were centrifuged and the supernatant was removed and transferred into deionized water. This process was repeated three times.

### 2.3. Preparation of the DNGHs

The microfragments were soaked in an aqueous solution containing 20 wt% NIPAM, 0.5 wt/wt% MBA and 1 μL mL^−1^ photoinitiator or 30 wt% AM, 0.5 wt/wt% MBA and 1 μL mL^−1^ photoinitiator for 24 hours. The soaked microfragments were centrifuged at 4500 rpm for 10 min and the supernatant was removed. The ink was cast into Teflon molds and crosslinked with UV light (Camag UV 4 Lamp) for 10 min to initiate the free radical polymerization reaction of the 2nd network.

### 2.4. Rheology

Rheological tests were performed using a DHR-3 TA Instrument with an 8 mm diameter parallel plate steel geometry. For the amplitude sweep measurements, strains from 0.01 to 200% at a constant frequency of 1 rad s^−1^ were employed. For the frequency sweeps, frequencies from 0.1 to 100 rad s^−1^ at 1% strain were applied. To characterize the shear thinning behavior, a flow sweep from 0.01 to 100 s^−1^ was performed. For stress relaxation experiments, the strain was altered between 1% and 100% for 65 seconds each. The cycle was repeated 5 times at a constant frequency of 1 rad s^−1^. For all measurements, the gap was kept constant at 1000 μm. All tests were performed on jammed microfragments.

### 2.5. Optical microscopy

The microgels were dispersed in water and put onto a glass slide. The microgels were observed with an optical microscope (Nikon Eclipse TS100).

### 2.6. Scanning electron microscopy (SEM)

The microfragments were frozen with liquid nitrogen and freeze-dried for 2 days with a freeze-dryer (Labconco, FreeZone 2.5). The freeze-dried microfragments were coated with 20 nm gold and palladium (quorum sputter coater). The SEM images were obtained using an SEM (Zeiss Merlin) equipped with HE-SE2 and operated with an acceleration voltage of 2 kV, a probe of current of 100 pA, and a working distance between 4 and 6 mm. The pores were measured using ImageJ.

### 2.7. Swelling ratio

The swelling ratio was calculated from bulk hydrogel cylinders. The weight (*W*) and the dimensions were measured as prepared and after swelling. The swelling ratio was calculated using the following equation:
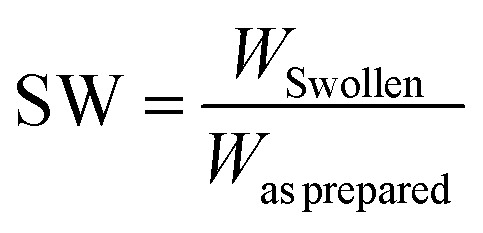


### 2.8. Tensile tests

Tensile tests were performed on dogbone samples with a cross-section of 5 × 2 mm^2^ with a Zwick Roell uniaxial testing machine with a 50 kN load cell. The samples were clamped and screwed tightly and stretched at a constant velocity of 100 mm min^−1^ until failure. Young's moduli were calculated from the slope of the stress–strain curve in a strain range of 5–15%.

The tensile tests at 60 °C were performed on dogbone samples with a cross-section of 5 × 2 mm^2^ on a Univert Cell scale uniaxial testing machine with a 5 kN load cell. The samples were heated to 60 °C prior to testing and then clamped. The container was closed and 60 °C water was added to maintain the temperature. The samples were stretched at a constant velocity of 100 mm min^−1^ until failure. Young's moduli were calculated from the slope of the stress–strain curve in a strain range of 5–15%.

### 2.9. Deswelling/reswelling

TDNGH rectangles were swollen overnight in deionized water, and 14 mm diameter circles were cut. The circular samples were placed in molds and submerged into a water bath of 70 °C. We chose 70 °C because this temperature is significantly above the LCST of PNIPAM, thereby ensuring that PNIPAM remains collapsed during the entire experiment. To reswell samples, the deswollen circular samples were put back to water at room temperature. The shrinkage and swelling were recorded as a function of time and the diameters were measured with ImageJ.

### 2.10. Preparation of bilayer samples

Bilayers were composed of a non-responsive and responsive layer. The first layer of non-responsive ink was cast into a rectangular Teflon mold (15 × 5 × 2 mm^3^) and exposed to UV light (Camag UV 4 Lamp) for 2 minutes. A 2 mm thick responsive DNGH 2nd layer was cast onto the previous layer and the full structure was exposed to UV for 10 minutes. After complete polymerization, the excess hydrogel was trimmed to create a 15 × 5 × 4 mm^3^ bilayer.

### 2.11. Actuation of bilayers

Bilayer samples were submerged from their as-cast state into a water bath of 75 °C. The top edge of the bilayer was secured with a needle to remain stationary. The bending actuation was recorded, and the angle (∠AOB) was measured as a function of time using ImageJ. Bilayers were stored in deionized water for use in applications.

### 2.12. 3D printing

The jammed ink was loaded into a 3 mL Luer-Lock syringe and centrifuged at 4300 rpm for 5 minutes to remove air. 3D printing was performed with a commercial 3D bioprinter (BioX, Cellink). The granular ink was extruded with a conical nozzle with a diameter of 250 μm and with a pressure driven piston. The ink was extruded at 10 mm s^−1^ and with a printing pressure of 29 kPa onto a glass substrate. The completed printed structures were crosslinked by exposure to UV light for 10 minutes to initiate the free radical polymerization reaction.

### 2.13. 3D printing of butterfly

A butterfly was 3D printed as a bilayer structure, with the first layer composed of a non-responsive ink containing jammed PAMPS microfragments, and the 2nd of responsive ink containing jammed PNIPAM microfragments. The non-responsive ink was 3D printed with a commercial bioprinter (BioX, Cellink) with the procedure described above and then exposed to UV light for 2 minutes. The responsive ink was then printed by hand using a syringe with a conical nozzle of 410 μm. The bilayer butterfly structure was placed under a UV lamp for 10 minutes to complete polymerization. The butterfly was actuated in a 70 °C water bath.

### 2.14. Butterfly CAD file

A butterfly CAD file was created using Fushion360 software based upon the original file designed by Lightshow74 as distributed by UltiMaker Thingiverse.

## 3. Results and discussion

### 3.1. Production of microfragments

**Fig. 1:**
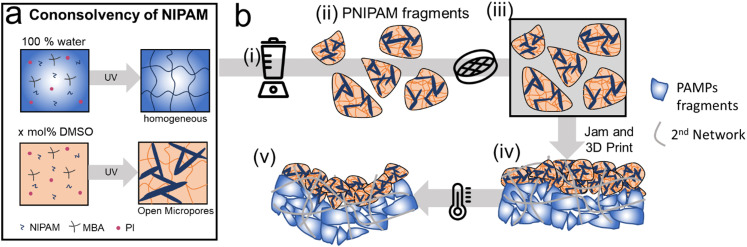
Schematic illustration of the production of porous microfragments. (a) The formation of PNIPAM in water results in homogeneous hydrogels whereas if formed in a mixture of DMSO and water, PNIPAM exhibits micropores with diameters of order of a few micrometers. (b) Schematic illustration of (i) the fragmentation of bulk hydrogels to produce (ii) microfragments that are (iii) filtered to remove particles with dimensions above 150 μm and subsequently soaked in an aqueous solution containing precursors for the 2nd network. The ink is cast or 3D printed before the (iv) 2nd network is formed to solidify the structure. (v) If heated above the LCST, the bilayer structure bends.

The response rate of PNIPAM is inherently slow because it relies on the diffusion of water. However, the response rate of PNIPAM increases if it contains open pores whose diameter is significantly larger than the mesh size of the hydrogel.^21,28^ To take advantage of this feature, we formulate microporous bulk PNIPAM by exploiting a solvent-induced phase separation. NIPAM is well soluble in pure water and pure DMSO. Its solubility strongly decreases if contained in a water and DMSO mixture.^38,39^ We leverage this behavior to trigger the phase separation of NIPAM-containing water into monomer-poor and monomer-rich phases. If NIPAM is dissolved in pure water, we expect it to attain a homogeneous structure that does not include any micrometer-sized pores. By contrast, if NIPAM is polymerized in water containing 40 or 60 mol% DMSO, we expect the resulting PNIPAM to contain connected micropores, as schematically illustrated in Fig. 1a.^38^

The precursor solution is Newtonian making it unsuitable for direct ink writing (DIW). To enable DIW, we fabricate bulk PNIPAM and fragment it using a blender,^40,41^ as shown in Fig. 1b. The resulting microfragments are filtered to remove particles with dimensions above 150 μm that risk clogging the printing nozzle. The sieved microfragments are washed in deionized water, as shown in Fig. 1b(ii). To firmly connect microfragments, we soak them in an aqueous solution containing acrylamide (AM), a crosslinker *N*,*N*′-methylenebis(acrylamide) (MBAA), and 2-hydroxy-2-methylpropiophenone as a photoinitiator, as shown in Fig. 1b(iii). The resulting reagent-loaded microfragments are jammed through centrifugation, 3D printed or cast before they are solidified through UV light illumination, as shown in Fig. 1b(iv). Thereby, we obtain thermo-responsive double network granular hydrogels (TDNGHs).^42^ The microstructured TDNGHs shrink if heated above the LCST. This volume change results in a bending if TDNGHs are cast or printed into an active layer that is connected to a non-responsive layer, as shown in Fig. 1b(v).

### 3.2. Morphology of responsive microfragments

**Fig. 2:**
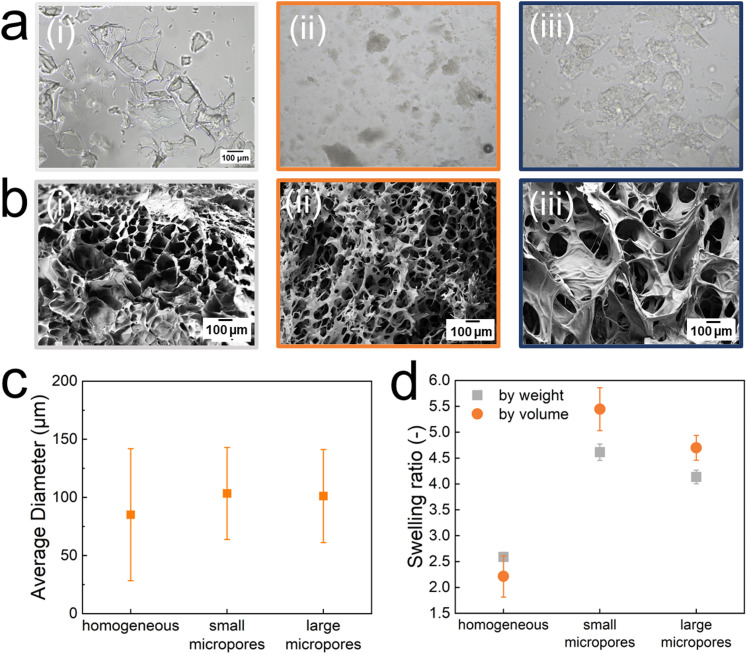
Visualization of homogeneous and microporous PNIPAM microfragments. (a) Optical images of (i) homogeneous and (ii) microporous microfragments formed in aqueous solutions containing 40 mol% and (iii) 60 mol% DMSO. (b) Scanning electron microscopy images of bulk homogeneous and microporous PNIPAM hydrogels. (c) The average diameter of the different types of microfragments (*n* = 100). (d) Swelling ratio of the homogeneous PNIPAM gel and samples with small and large micropores by weight (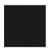 grey) and volume (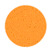 orange). The sample possessing small micropores display the highest swelling ratio.

All tested microfragments possess poorly defined shapes and broad size distributions, as shown in Fig. 2a, in good agreement with the literature.^40,43,44^ In the hydrated state, PNIPAM produced from aqueous solutions is transparent, as shown in the optical micrograph in Fig. 2a(i). The contrast is much higher if PNIPAM has been produced within a DMSO–water mixture, as shown in Fig. 2b(ii) and (iii). These results suggest that PNIPAM produced in a DMSO–water mixture includes pores of order of the wavelength of visible light such that they scatter visible light. To test this suggestion, we visualize bulk gels with a scanning electron microscope (SEM), as shown in Fig. 2b. To minimize the risk for sample preparation artifacts, we freeze dry the hydrogels. All tested bulk hydrogels display pores, in good agreement with previous reports.^45^ The pore morphology depends on the DMSO content contained in the precursor solution: samples produced in the presence of 40 mol% DMSO exhibit smaller micropores than samples produced from solutions containing 60 mol% DMSO. The sizes of these pores are too small to reliably quantify them with confocal microscopy or microcomputed tomography. Hence, we quantify the pores in the dried state using SEM. Samples produced in the presence of 40 mol% DMSO have an average diameter of dried pores of (2.2 ± 1.2) μm. By contrast, samples produced in the presence of 60 mol% DMSO have an average diameter of dried pores of (4.6 ± 2.5) μm, as shown in Fig. S1 (ESI†). Note that pores are significantly larger in the hydrated state. Because we cannot reliably quantify the size of hydrated pores, we refer to micropores included in PNIPAM produced from solutions containing 40 mol% DMSO as small ones and those included in PNIPAM made from solutions containing 60 mol% DMSO as large ones.

To ensure that the printing process remains robust and prevent large microfragments from clogging the nozzle, we remove particles exceeding 150 μm in size through filtration. We quantify the diameter of the filtered microfragments from optical micrographs by measuring their diagonal. All investigated hydrated microfragments display an average diagonal between 85 and 105 μm, as shown in Fig. 2c. The swelling ratio of the microfragments determines the amount of precursor for the second network that can be loaded into them. Given the importance of this parameter for the mechanical properties of TDNGHs, we quantify it. Homogeneous bulk hydrogels that do not possess any micropores swell least and those possessing small micropores swell most, as shown in Fig. 2d, in good agreement with previous reports.^21^

### 3.3. Rheological properties and printability of jammed microfragments

**Fig. 3:**
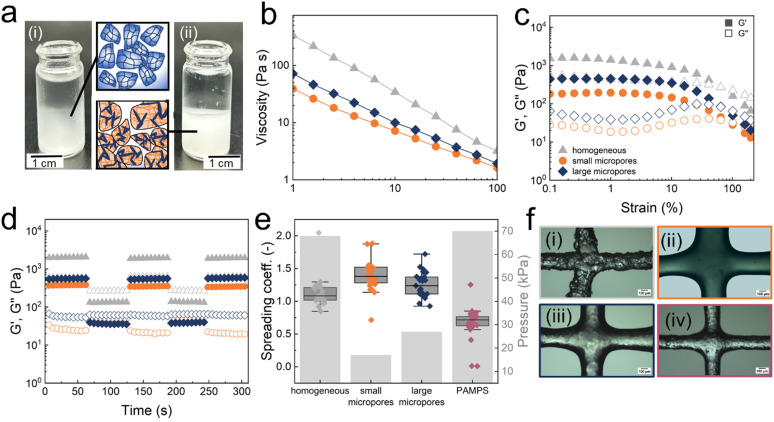
Rheological properties and printability of jammed PNIPAM microfragments. (a) Photograph of jammed PNIPAM fragments that (i) are homogeneous and (ii) contain small micropores. (b) Frequency, (c) amplitude sweeps, and (d) shear recovery curves of jammed fragments that are homogeneous (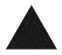 grey) and contain small (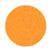 orange) and large micropores (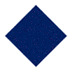 blue). (e) Printability of the jammed fragments. The spreading coefficient as a function of the porosity of microfragments (*n* = 25). The minimum pressure required to print the jammed microfragments is shown on the right. The best shape fidelity is achieved with homogeneous PNIPAM and PAMPS fragments. (f) Optical micrographs of printed grids composed of jammed PNIAPM microfragments (i) that are homogeneous (ii) contain small, and (iii) large micropores and (iv) homogeneous PAMPS microfragments.

Inks suitable for DIW must be shear thinning, possess a low flow stress to enable printing at moderate pressures and display a fast stress recovery. Inks composed of jammed hydrogel-based microparticles and microfragments typically fulfil these rheological requirements.^41,44,46^ To enable direct ink writing of our microfragments, they are jammed through centrifugation, as shown in Fig. 3a.^46,47^ Indeed, all tested inks are shear thinning, as shown in Fig. 3b. They display a linear viscoelastic region at strains from 0.1 to 80% and their flow stress is between 38 Pa for inks composed of jammed microfragments with large micropores and 419 Pa for those made of jammed homogeneous counterparts, as shown in the amplitude sweep in Fig. 3c. Moreover, they all display a fast stress recovery, as shown in Fig. 3d. These rheological properties are similar to those of the more commonly used inks composed of jammed nonresponsive homogeneous poly(2-acrylamido-2-methyl-1-propanesulfonic acid) (PAMPS) microfragments, as shown in Fig. S2 (ESI†). This similarity indicates that our inks are well suited for DIW, in stark contrast to most PNIPAM systems derived from bulk solutions.^9,10^

To quantify the printing resolution, we measure the spreading coefficient of the different inks. The spreading coefficient of our inks varies between 0.75 and 2.1. The lowest spreading coefficient and highest printing pressure are measured for inks composed of jammed homogeneous fragments, as summarized in Fig. 3e and illustrated with the optical micrographs in Fig. 3f. The ink composed of jammed non-responsive PAMPS microfragments displays a similar spreading coefficient and flow point as that composed of jammed homogeneous PNIPAM microfragments, as shown in Fig. 3e. These results indicate that the spreading coefficient and yield stress mainly depend on the micropores within microgels. Because we keep the overall monomer concentration constant, the introduction of micropores results in a concentration of reagents within the monomer-rich phase, which translates into a stiffening of the microfragments.

### 3.4. Influence of the microfragments on the mechanical properties of DNGHs

Granular hydrogels are typically soft because of weak inter-particle interactions. The stiffness of granular hydrogels can be increased if they are connected through a 2nd hydrogel network that interpenetrates and covalently crosslinks them, resulting in double network granular hydrogels (DNGHs).^42,48^ To solidify our TDGNHs, we soak the microfragments in an aqueous solution containing 20 wt% AM and 0.5 wt/wt% crosslinker. The reagent-loaded microfragments are jammed, cast into dogbone-shaped Teflon molds, and solidified by exposing the samples to UV light to initiate the free radical polymerization of the precursors contained in the microfragments. We subsequently wash TDNGHs with water to remove DMSO, as indicated by the absence of any DMSO peak in the FTIR spectra, shown in Fig. S3 and detailed in the ESI.† As a result of the compositional similarity of all tested TDNGHs, they exhibit the same cloud point temperature, as shown in Fig. S4 (ESI†).

**Fig. 4:**
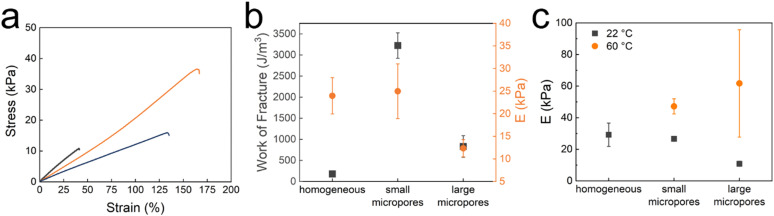
Influence of microfragments on the mechanical properties of DNGHs. (a) Stress–strain curves of TDNGHs fabricated from PNIPAM microfragments that are homogeneous (grey) and contain small (orange) and large micropores (blue). PAM is used as a 2nd network. (b) Work of fracture (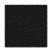 grey) and Young's Modulus (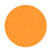 orange) of TDNGHs made of microfragments that are homogeneous and contain small and large micropores (*n* = 3). (c) Young's moduli of TDNGHs measured at 22 °C (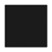 grey) and 60 °C (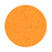 orange) (*n* = 3).

To assess the impact of the micropores within the microfragments on the mechanical properties of TDNGHs, we conduct tensile tests on them, as shown in Fig. 4a. TDNGHs composed of microporous microfragments display a higher work of fracture, calculated as the area under the stress–strain curve,^49^ than those made of homogeneous microfragments that do not include any micropores, as shown in Fig. 4b. For example, the work of fracture of TDNGHs produced with microfragments with large micropores is 4.5-fold higher than that of TDNGHs made from homogeneous microfragments. The work of fracture increases even more, 18-fold, if TDNGHs are made of microfragments with small micropores, as shown in Fig. 4b. We assign this strong increase in the work of fracture to the higher swelling ratio of microporous microfragments, which hence can uptake a larger amount of precursors for the 2nd network, resulting in a denser 2nd network within TDNGHs, as shown in Fig. 2d. Remarkably, the introduction of small micropores into microfragments does not significantly change the Young's modulus of TDNGHs, as shown in Fig. 4c. By contrast, larger micropores reduce the Young's modulus 2-fold, as shown in Fig. 4c. These results suggest that the microporosity of microfragments mainly increases the toughness of TDNGHs.

The toughness of DNGHs is governed by that of the 2nd network.^42,50^ To test the influence of the 2nd network on the mechanical properties of TDNGHs, we soak microfragments in an aqueous solution containing 30 wt% AM and 1 wt/wt% MBA. Increasing the monomer concentration in the precursor solution of the 2nd network increases the Young's modulus of TDNGHs to 26 kPa and decreases the work of fracture to 2536 J m^−3^, as shown in Fig. S5 (ESI†). Yet, the influence of the microfragment morphology on the mechanical properties of TDNGHs remains the same: the work of fracture of TDNGHs is highest if they are made of microfragments containing small micropores, as shown in Fig. S5b (ESI†).

To assess the influence of the composition of the 2nd network on the work of fracture of TDNGHs, we exchange PAM with PNIPAM. To achieve this goal, we soak microfragments in an aqueous solution containing 20 wt% NIPAM and 0.5 wt/wt% MBA. The work of fracture decreases 5.6-fold from 3223 J m^−3^ to 567 J m^−3^ if PNIPAM is used as a 2nd network, as shown in Fig. S6a (ESI†). We assign the much lower work of fracture to the lower work of fracture of PNIPAM compared to that of PAM.^30^ By analogy, the Young's modulus of TDNGHs possessing PNIPAM as a 2nd network decreases from 25 kPa to 19 kPa, as shown in Fig. S6 (ESI†). Yet, the influence of micropores within microfragments on the mechanical properties of TDNGHs remains the same. The work of fracture of TDNGHs possessing PNIPAM as a 2nd network increases almost 7-fold and the Young's modulus 1.8-fold if they are composed of microfragments with small micropores compared to those composed of homogeneous microfragments, as shown in Fig. S6 (ESI†).

To assess the influence of temperature on the mechanical properties of TDNGHs possessing PAM as a 2nd network, we perform tensile tests at 60 °C, a temperature well above the LCST of PNIPAM. As expected, Young's modulus of TDNGHs composed of microfragments possessing small micropores at 60 °C is two-fold higher than that at 22 °C, as shown in Fig. 4c. Note that TDNGHs made of homogeneous microfragments become brittle if heated above the LCST. The brittleness of these samples prevents a reliable testing of their mechanical properties at temperatures exceeding their LCST.

### 3.5. Thermo-responsiveness of TDNGHs

**Fig. 5:**
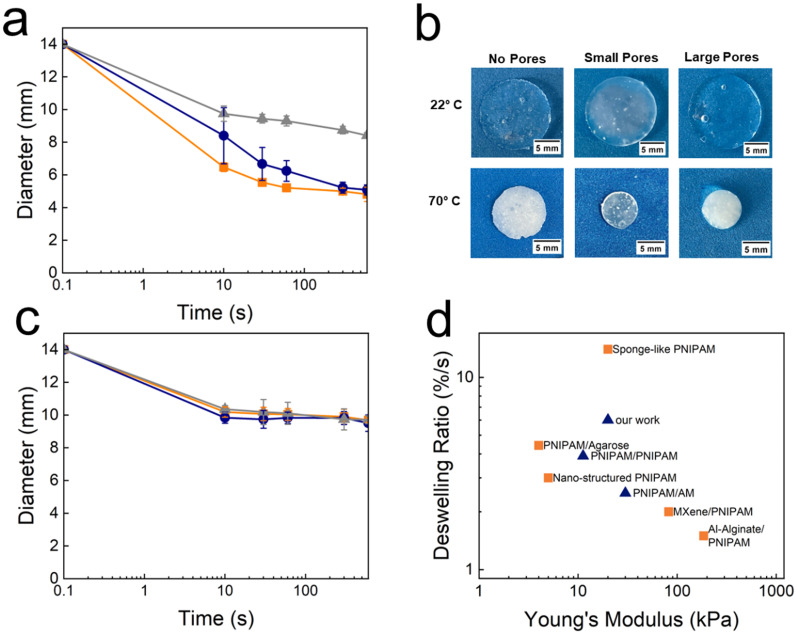
Temperature-induced deswelling of TDNGHs. (a) Deswelling of TDNGHs fabricated from PNIPAM microfragments that are homogeneous (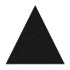 grey) and contain small (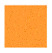 orange) and large micropores (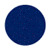 blue) connected by a PNIPAM 2nd network as a function of time at 70 °C. The highest deswelling rate is achieved within samples containing microporous PNIPAM microfragments. (b) Photographs of TDNGHs at 22 °C and 70 °C. (c) Deswelling of TDNGHs fabricated with PNIPAM microfragments that are homogeneous (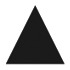 grey) and contain small (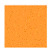 orange) and large micropores (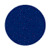 blue) connected through a PAM 2nd network as a function of time kept at 70 °C. (d) Ashby plot of normalized deswelling rates as a function of the Young's modulus of PNIPAM-based hydrogels reported in the literature.^11,21,55–57^ Blue symbols represent DIW 3D printable systems and orange symbols represent non-printable counterparts.

To assess the thermo-responsiveness of TDNGHs, we cast and cut circular disks with a diameter of 14 mm and a thickness of 2 mm and incubate them in a water bath at 70 °C, as shown in the photograph in Fig. S7 (ESI†). As expected, the disks deswell at 70 °C. To assess the influence of the microfragment morphology on the deswelling kinetics, we quantify the time-dependent change in the disk diameter. TDNGHs made of microfragments containing small micropores possess the fastest response rate. The diameter of these samples changes during the first 10 s by 0.75 mm s^−1^, corresponding to a relative diameter change of 10.74% s^−1^ and levels off thereafter, as shown in Fig. 5a. TDNGHs composed of microfragments possessing large micropores deswell slower; their diameter reduces by 0.56 mm s^−1^, corresponding to a relative diameter change of 5.4% s^−1^ whereas the diameter of the samples containing homogeneous microfragments decreases by 0.43 mm s^−1^, corresponding to a relative diameter decrease of 2.5% s^−1^, as shown in Fig. 5b. In line with this trend, the diameter of bulk PNIPAM, that is primarily used for thermo-responsive actuation in soft robots, decreases even slower, and the relative diameter changes by 0.13% s^−1^.^51^ These results indicate that interconnected micropores increase the deswelling rate, most likely because they increase the degree of swelling of these hydrogels, thereby facilitating deswelling.

Our results indicate that the deswelling speed of TDNGHs is closely related to the swelling degree of the microfragments they are made from. We expect the swelling degree to influence the extent of the response of TDNGHs. To test this expectation, we quantify the extent of the response of TDNGHs by measuring the diameter of the as-prepared TDNGH disks and that of samples that have been soaked in water at room temperature for at least 24 hours. TDNGHs possessing PNIPAM as a 2nd network exhibit a higher swelling ratio compared to those containing PAM as a 2nd network, as shown in Fig. S8 (ESI†). We hypothesize that this is due to the higher swelling ratio of PNIPAM hydrogels comparted to that of PAM hydrogels.^52,53^ To test the reversibility of the deswelling, we cool them to room temperature, as shown in Fig. S9 (ESI†). The reswelling is more time consuming: it takes approximately 4 hours until equilibrium is reached because the reverse process is diffusion limited.^51^ The micropore-induced acceleration of the deswelling kinetics is in good agreement with previous reports.^54^ Yet, in previously reported thermo-responsive systems, the response rate had to be traded off with the stiffness of the material or the LCST. By contrast, the response rates of TDNGHs can be increased without affecting their stiffness or LCST, as shown in Fig. 4. This result highlights the benefit of the granular structure.

Our results indicate that we can increase the deswelling speed without compromising the stiffness of TDNGHs if we employ porous microgels. To test if we can combine the fast deswelling with good mechanical properties, we replace the PNIPAM 2nd network with a non-thermo-responsive PAM network. As expected, TDNGHs containing PAM as a 2nd network deswell slower and to a lesser extent compared to those containing PNIPAM as a 2nd network, as shown in Fig. 5c and Fig. S9 (ESI†). Yet, their work of fracture is 10-fold higher than that of TDNGHs containing PNIPAM as a 2nd network. Moreover, the deswelling speed of these load-bearing TDNGHs is similar to that of previously reported PNIPAM formulations that were rather weak. The combination of reasonably high stiffness, fast actuation speed and unchanged LCST achieved by porous TDNGHs opens up new applications in soft robotics that simultaneously require fast response rates and load-bearing properties, as summarized in the Ashby plot in Fig. 5d.

### 3.6. Bending of double layer hydrogels

**Fig. 6:**
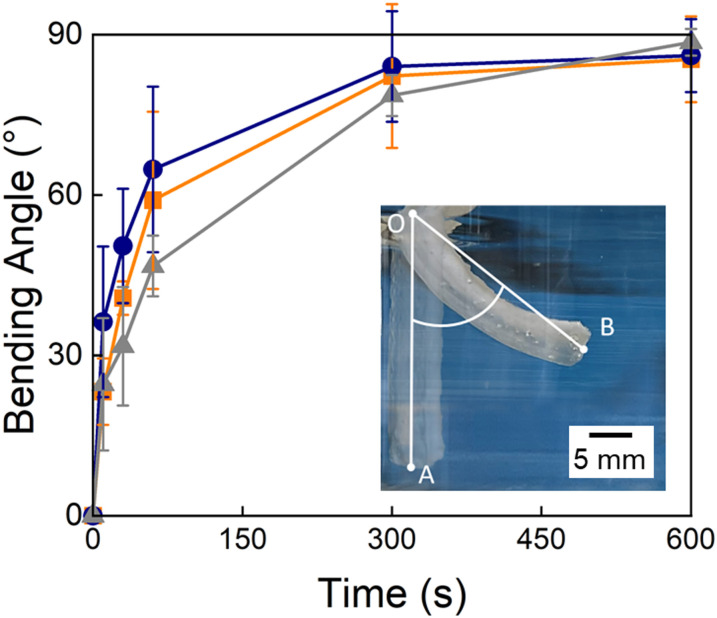
Degree of actuation of TDNGHs. The bending angle as a function of time after the bilayers have been actuated in a water bath of 70 °C. The active layer is composed of TDNGHs made of microfragments that are homogeneous (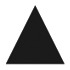 grey) and contain small (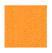 orange) and large (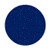 blue) micropores. The inset is a photograph encompassing the definition of the bending angle, ∠AOB.

To assess the suitability of our TDNGHs as thermo-responsive actuators, we cast a bilayer composed of a 2 mm thick active layer made of PNIPAM microfragments containing small micropores and a 2 mm thick inert layer composed of PAMPS microfragments. If incubated at elevated temperatures, this structure bends because the active PNIPAM-based layer deswells whereas the inactive layer does not. As a result of the deswelling, the active layer becomes opaque, while the passive layer remains transparent. To determine the extent of actuation, we record time-lapse images and quantify the bending angle as exemplified in the inset in Fig. 6. Samples whose active layer is composed of PNIPAM microfragments reach a bending angle of 90° after 10 minutes, irrespective of the presence of micropores in the fragments. We assign the similar behavior of the samples to the inert layer, that is identical and delays their bending, thereby minimizing the effect of micropores within the active layer.

### 3.7. Applications of thermo-responsive double layer hydrogels

**Fig. 7:**
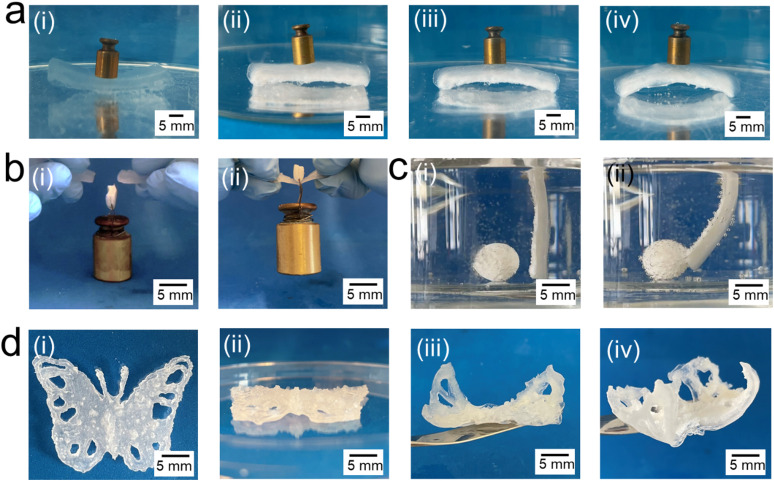
Proof-of-concept of thermally actuated TDNGHs. (a) (i) Bilayer composed of an inert layer made of PAMPS microfragments and a responsive layer composed of PNIPAM microfragments with small micropores. (ii) If immersed in an aqueous solution at 60 °C, the bilayer starts to bend after 10 minutes to lift a 2 g weight after (iii) 30 and (iv) 50 minutes. (b) A TDNGH made with PNIPAM microfragments with small micropores at (i) room temperature, where it breaks when loaded with 50 g and (ii) at 70 °C where it holds the load that constitutes 50-times the weight of the sample itself. (c) Bilayer DNGHs immersed in water at 70 °C after (i) 0 and (ii) 30 minutes when the 3D printed ball is kicked to the left due to bending. (d) 3D printed butterfly consisting of a bilayer. The inert DNGH is made of PAMPS microfragments in a PAM 2nd network, and the active layer is made of PNIPAM microfragments with small micropores connected with a PNIPAM 2nd network (i top view and ii side view) after printing at room temperature and (iii and iv) immersed in water at 70 °C where it starts to bend the wings after 10 minutes.

To demonstrate the potential of TDNGHs as soft actuators, we fabricate a bilayer composed of an inert 2 mm thick DNGH made of PAMPS microfragments connected through a PAM network and a 2 mm thick TDNGH composed of PNIPAM microfragments containing small micropores connected through a PNIPAM network. When this bilayer is placed in a water bath at 70 °C, it bends, thereby lifting a 2 g weight, which constitutes 85% of its bodyweight, as shown in Fig. 7a(ii)–(iv). We harness the responsiveness of this material to push a 3D printed ball at elevated temperatures, as demonstrated in Fig. 7c and Movie M1 (ESI†).

To illustrate the temperature-dependent mechanical properties of our TDNGHs, we fabricate a TDNGH with a cross-section of 10 × 2 mm^2^. At room temperature, this sample breaks if loaded with a 50 g weight, as shown in Fig. 7b(i); by contrast, if we perform the same test at 70 °C, the TDNGH lifts this weight, which is more than 50-fold heavier than the weight of the sample itself, as shown in Fig. 7b(ii).

An important advantage of our system is its 3D printability through direct ink writing, which enables the fabrication of intricate 3D structures. We exploit this feature to 3D print a wing-bending butterfly, as shown in Fig. 7d. The butterfly is composed of an inactive DNGH layer made of PAMPS microfragments and an active layer containing PNIPAM microfragments. If incubated at 70 °C, the active layer deswells, such that the wings bend despite of its heterogeneous structure, as shown in Fig. 7d(ii) and (iv). These examples demonstrate the potential of TDNGHs for thermally induced soft actuating applications.

## 4. Conclusion

We introduce 3D printable thermo-responsive double network granular hydrogels composed of PNIPAM microfragments encompassing micropores that are connected through a 2nd PNIPAM network. We demonstrate that the deswelling rate of TDNGHs depends on the microporosity of the microfragments and the composition of the 2nd network. The micropores within the microfragments add an additional benefit: they increase Young's modulus of TDNGHs up to 1.8-fold and their work of fracture up to 18-fold compared to TDNGHs made from homogeneous counterparts. We foresee the good mechanical properties of TDNGHs combined with their thermo-responsiveness and 3D printability to open up new possibilities to thermally actuate soft robots with additional work also on scaffolds used for tissue engineering.

## Conflicts of interest

The authors declare no conflicts of interest.

## Supplementary Material

MA-006-D5MA00511F-s001

MA-006-D5MA00511F-s002

## Data Availability

Data will be made available upon request.
