# Supplementary material for: Microstructured thermo-responsive double network granular hydrogels
Source: Mater Adv. 2025 Jun 16;6(15):5089–99. doi: 10.1039/d5ma00511f (PMC12186764; doi:10.1039/d5ma00511f)
Supplement: MA-006-D5MA00511F-s002 [file MA-006-D5MA00511F-s002.pdf]

Supplementary Information (SI) for Materials Advances.
This journal is © The Royal Society of Chemistry 2025

Supporting Information

Microstructured Thermo-responsive Double Network Granular Hydrogels

*Alexandra Thoma, Reece Whatmore and Esther Amstad**

Movie M1: A bilayer composed of a responsive and non-responsive layer pushes forward a 3D printed ball underwater at elevated temperatures within 30 min.

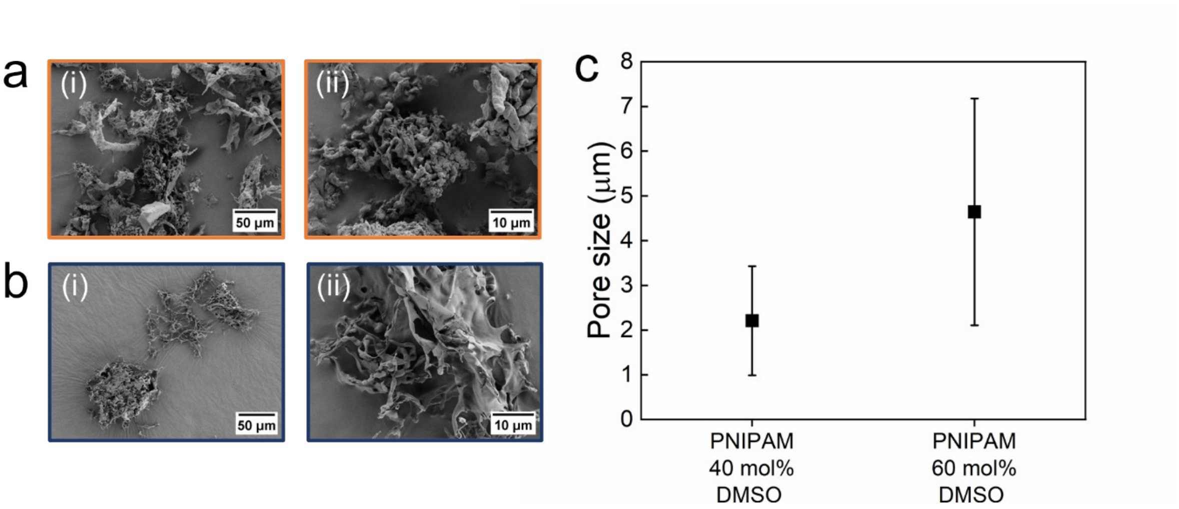

Figure S1. Diameter of micropores of PNIPAM polymerized in a solution containing 40 mol% DMSO and 60 mol% DMSO. (a and b) Scanning electron microscopy images of (b) microporous microfragments formed in aqueous solutions containing 40 mol% and (c) 60 mol% DMSO at (i) lower and (ii) higher magnification. (c) The diameters are measured on freeze dried samples (n = 50). PNIPAM polymerized in a solution containing 60 mol% DMSO contain larger micropores compared to that produced in a solution containing 40 mol% DMSO.

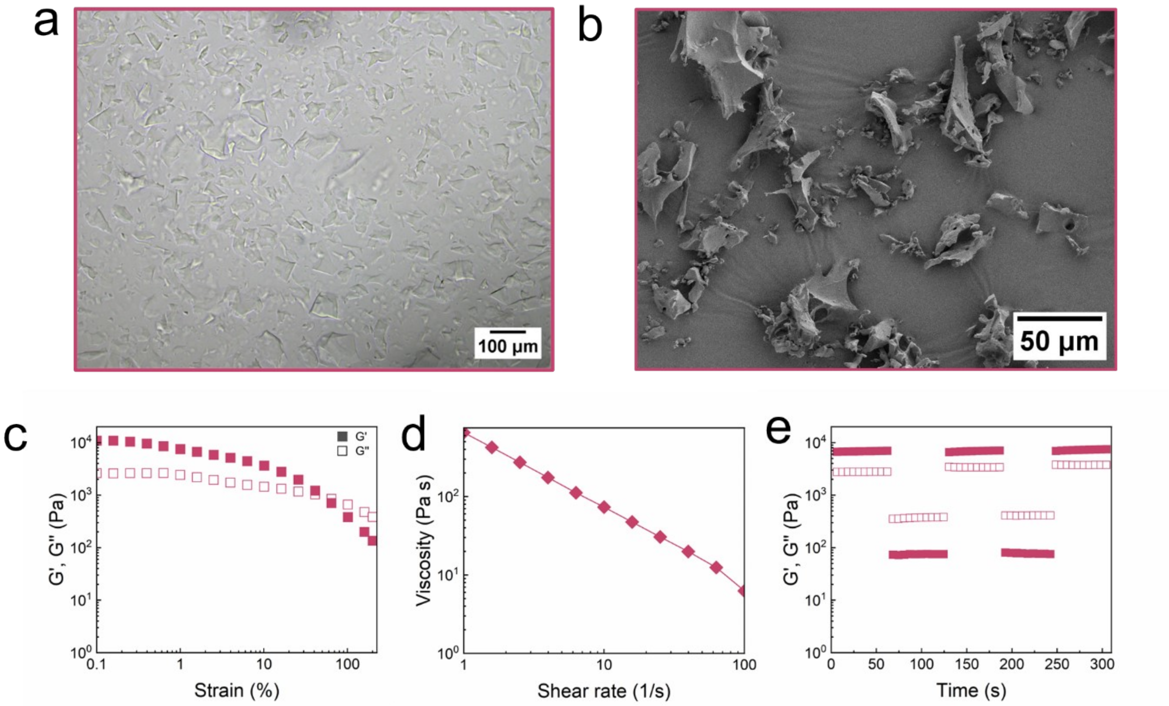

Figure S2. (a) Optical and (b) scanning electron microscopy images of PAMPS microfragments. (c) Amplitude, (d) frequency sweeps and (e) shear recovery measurement of jammed PAMPS microfragments.

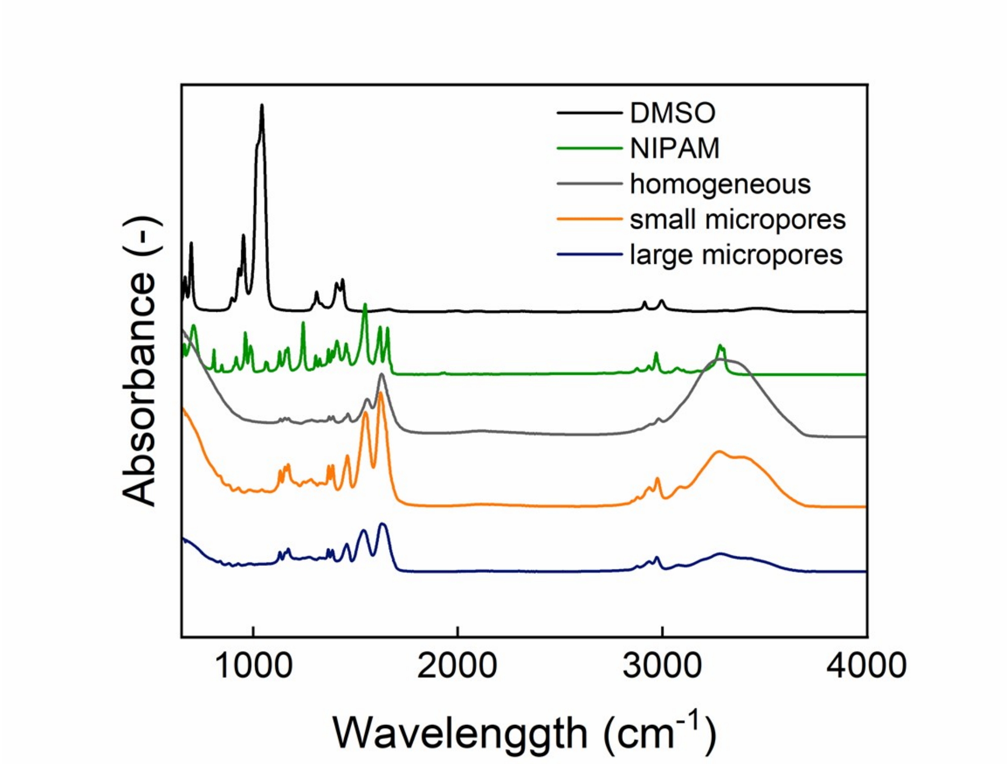

Figure S3: FTIR spectra of DMSO, PNIPAM and DNGHs that are homogeneous, contain small and large micropores, indicating that our washing protocol fully removes DMSO from

TDNGHs. Moreover, these spectra demonstrate that the composition of all PNIPAM microfragments is identical.

FTIR analysis shows that the DMSO peak at 1044 cm^{-1} disappears in all TDNGH samples, indicating the effective removal of DMSO during washing with deionized water. The spectra also reveal a peak at 2970 cm^{-1}, characteristic of the CH_3 stretch vibration of PNIPAM, and a peak at 1550 cm^{-1}, attributed to the C=O stretch vibration. Additionally, the peak at 3292 cm^{-1}, corresponding to the N-H stretch vibration, is observed in all TDNGH samples. These findings confirm that the composition of the TDNGHs are identical.

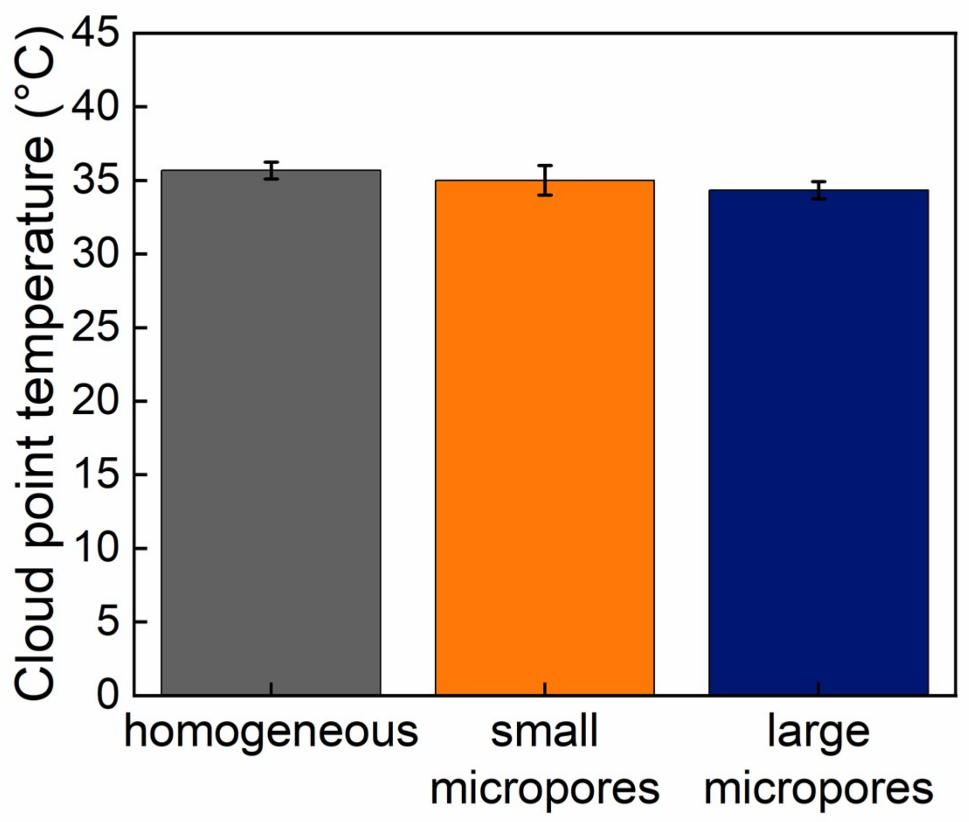

Figure S4. Cloud point temperatures of TNDGHs with fragments that are homogeneous, contain small and large micropores.

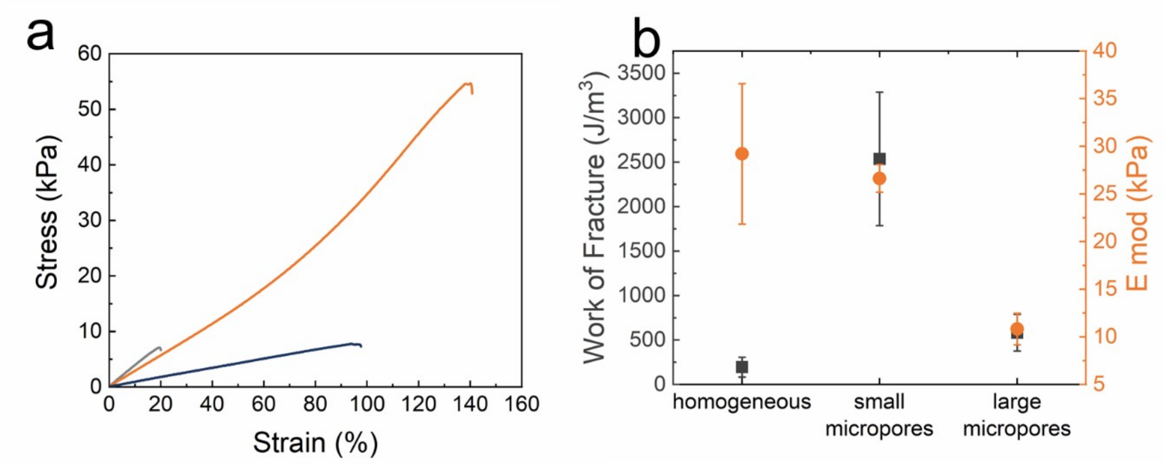

Figure S5. Influence of micropores within microfragments connected by a PAM 2nd network containing 1 wt/wt% crosslinker on the mechanical properties of TDNGHs. (a) Stress-strain curves of TDNGHs fabricated from PNIPAM microfragments that are homogeneous (grey), contain small (orange) and large micropores (blue). (b) Work of fracture (■ grey) and Young's Modulus (■orange) as a function of the micropores within PNIPAM microfragments.

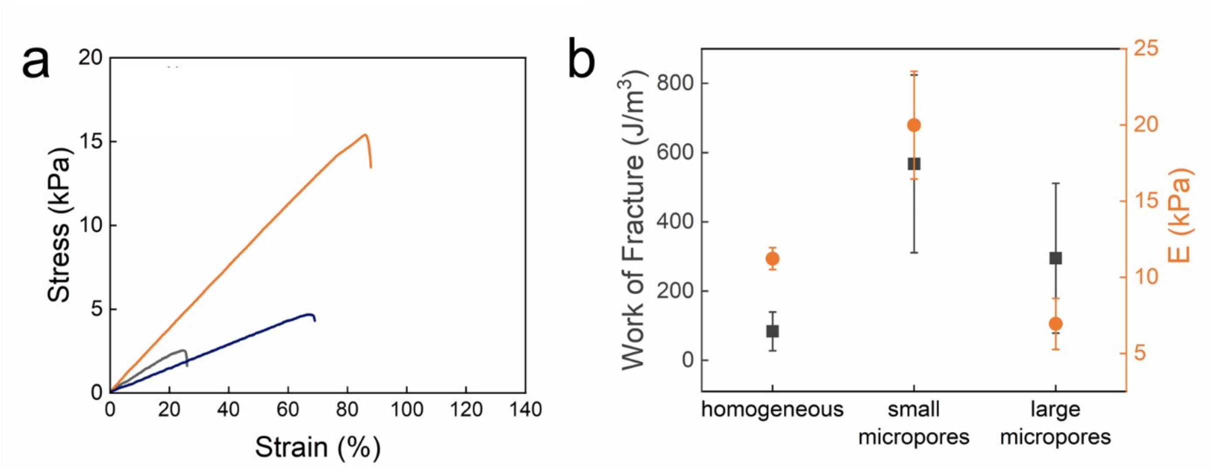

Figure S6. Influence of the microfragment morphology on the mechanical properties of TDNGHs possessing PNIPAM as a 2nd network. (a) Stress-strain curves of TDNGHs fabricated from PNIPAM microfragments that are homogeneous (grey), contain small (orange) and large micropores (blue) connected by a PNIPAM network made from an aqueous solution containing 20 wt% NIPAM and 0.5 wt/wt% crosslinker. (b) Work of fracture (■ grey) and Young's Modulus (● orange) as a function of the micropores within microfragments.

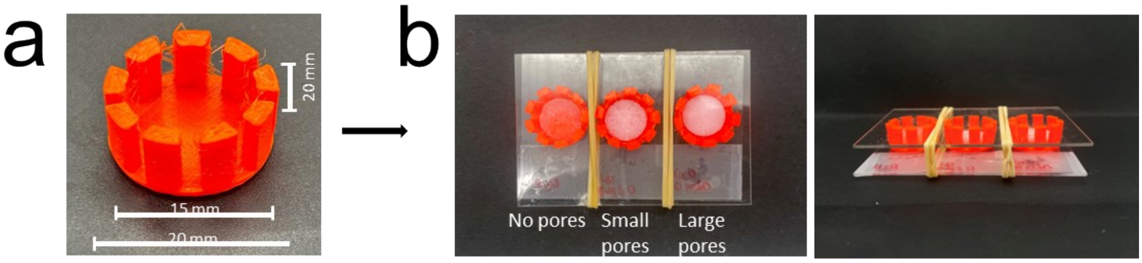

Figure S7. (a) Photograph 3D printed container of TDNGHs used to quantify (b) the deswelling kinetics.

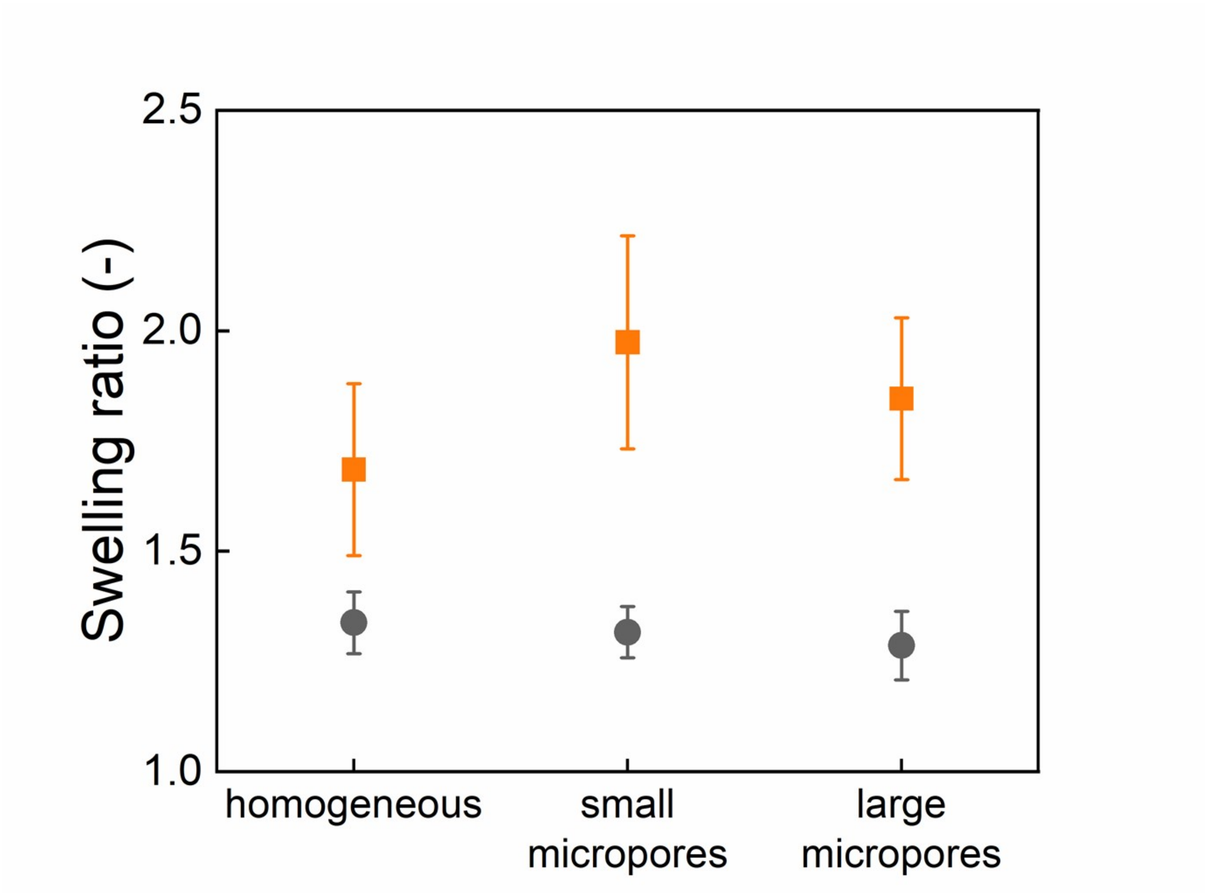

Figure S8. Swelling ratio of TDNGHs containing PNIPAM (orange) and PAM (grey) as a 2nd network. The TDNGHs containing microfragments with small micropores soaked in deionized water swell to an almost two-fold volume compared to that of the as prepared state.

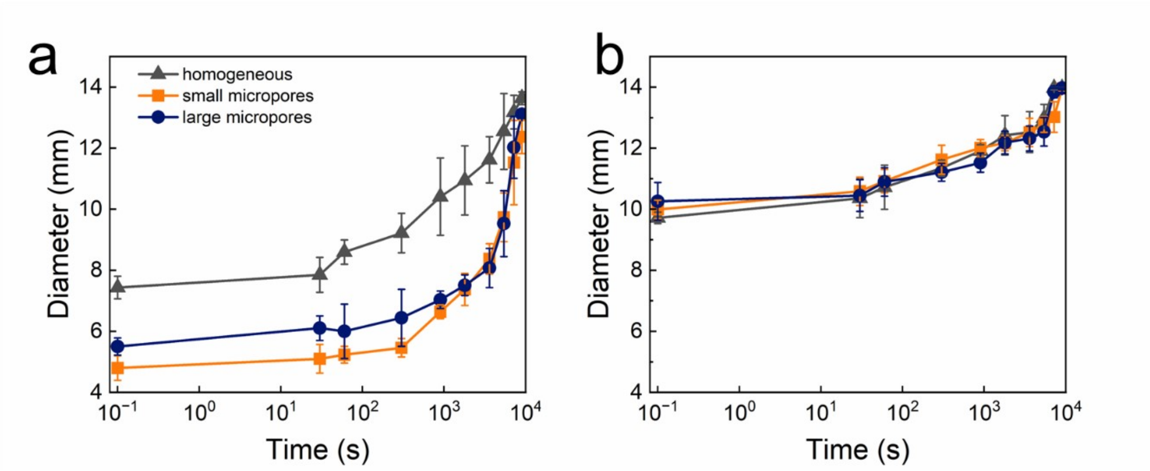

Figure S9. Reswelling of deswollen TNDGHs at room temperature. (a) Reswelling of TDNGHs fabricated from PNIPAM microfragments that are homogeneous (▲ grey), contain small (■ orange) and large micropores (● blue) connected by a PNIPAM 2nd network. Samples have been exposed to 70°C for 10 min before being cooled to room temperature for the indicated time. (b) Deswelling of TDNGHs fabricated with PNIPAM microfragments that are homogeneous (▲ grey), contain small (■ orange) and large micropores (● blue) connected through a PAM 2nd network. Samples have been exposed to 70°C for 10 min before being cooled to room temperature for the indicated time.
